# STORIES OF TRAUMA AND RECONCILIATION OF WORLD WAR II VETERANS

**DOI:** 10.1093/geroni/igz038.2822

**Published:** 2019-11-08

**Authors:** Hanna K Ulatowska, Tricia Santos, Diane Walsh, Jilliane Lagus, Mitchell Pruett, Sara Aguilar

**Affiliations:** The University of Texas at Dallas, Dallas, United States

## Abstract

The present qualitative study examined the reconciliation of trauma experienced by 55 World War II veterans (22 aeronautical crew members, 27 non-pilot combatants, and 6 veterans with dementia) demonstrated via testimonial language within a semi-structured interview. The research team considered themes of language coherence as they relate to veteran experiences of trauma and reconciliation. Trauma literature documents the importance of personal narratives in both identifying and reconciling traumatic experiences. This study examined morals and values of participants, traumatic experiences either lived or witnessed, and reconciliation of trauma as demonstrated by the coherence of participants’ linguistic and paralinguistic communication. Linguistic analysis included the use of evaluative and emotional language; linguistic devices such as crowding, topic maintenance, and humor; and lessons learned from trauma and the reconciliation process. Prosody was analyzed as a paralinguistic indicator of trauma and reconciliation using audio recordings of semi-structured interviews. The primary findings revealed that highly coherent language is present among participants with distinct content when comparing episodes from youth and reflections of experience in old age. The unique differences demonstrated overall strength of veterans’ narrative identity throughout their lives. Strength of identity and coherence of language indicated adequate reconciliation of traumatic events. Reconciliation of trauma was also evident in veterans’ participation in the study and generative behavior described in testimonial language.

